# *﻿Baiyuerius* gen. nov., a new genus of Coelotinae (Araneae, Agelenidae) spiders from China and Vietnam

**DOI:** 10.3897/zookeys.1165.101946

**Published:** 2023-05-30

**Authors:** Zhe Zhao, Bing Li, Xiaoqing Zhang, Francesco Ballarin, Dinh-Sac Pham, Shuqiang Li

**Affiliations:** 1 Institute of Zoology, Chinese Academy of Sciences, Beijing 100101, China Institute of Zoology, Chinese Academy of Sciences Beijing China; 2 College of Life Sciences, Langfang Normal University, Langfang, Hebei 065000, China Langfang Normal Univrsity Langfang China; 3 Systematic Zoology Laboratory, Department of Biological Sciences, Graduate School of Science, Tokyo Metropolitan University, 1-1 Minami Osawa, Hachioji-shi, Tokyo, 192-0397, Japan Tokyo Metropolitan University Tokyo Japan; 4 Department of Zoology, Museo di Storia Naturale of Verona, Lungadige Porta Vittoria, 9, I-37129 Verona, Italy Museo Civico di Storia Naturale of Verona Verona Italy; 5 Vietnam National Museum of Nature (VNMN), Vietnam Academy of Science and Technology (VAST), 18 Hoang Quoc Viet, Cau Giay, Hanoi, Vietnam Vietnam Academy of Science and Technology Hanoi Vietnam

**Keywords:** Asia, DNA barcoding, new species, phylogeny, taxonomy

## Abstract

*Baiyuerius***gen. nov.**, a new genus of the subfamily Coelotinae F. O. Pickard-Cambridge, 1893 is described, including five new species: *B.daxi***sp. nov.** (♀), *B.pindong***sp. nov.** (♂), *B.tamdao***sp. nov.** (♀), *B.zhuping***sp. nov.** (♂) and *B.zuojiang***sp. nov.** (♂♀), from southern China and northern Vietnam. Our molecular phylogenetic analyses support *Baiyuerius* gen. nov. as monophyletic and as a sister group of the newly established genus *Yunguirius* Li, Zhao & Li, 2023.

## ﻿Introduction

Agelenidae C. L. Koch, 1837 is one of the ten largest spider families in the world, including 1377 species in 94 genera. In particular, the subfamily Coelotinae F. O. Pickard-Cambridge, 1893 is exceptionally diverse with 801 species in 39 genera (WSC 2023). This subfamily is widely distributed, occurring in Asia, Europe, and North America. In the last decade, 16 genera have been established to accommodate the Asian coelotine species: *Aeolocoelotes* Okumura, 2020 (Japan); *Curticoelotes* Okumura, 2020 (Japan); *Dichodactylus* Okumura, 2017 (Japan); *Flexicoelotes* Chen, Li & Zhao, 2015 (China); *Griseidraconarius* Okumura, 2020 (Japan); *Guilotes* Zhao & Li, 2018 (China); *Hengconarius* Zhao & Li, 2018 (China); *Jishiyu* Lin & Li, 2023 (China); *Nesiocoelotes* Okumura & Zhao, 2022 (Japan); *Nuconarius* Zhao & Li, 2018 (China); *Papiliocoelotes* Zhao & Li, 2016 (China); *Sinocoelotes* Zhao & Li, 2016 (China, Thailand); *Sinodraconarius* Zhao & Li, 2018 (China); *Troglocoelotes* Zhao & Li, 2019 (China); *Vappolotes* Zhao & Li, 2019 (China); *Yunguirius* Li, Zhao & Li, 2023 (China) (Chen et al. 2015, 2016; Zhao and Li 2016; Okumura 2017, 2020; Li et al. 2018a, b, c, 2019a, b, 2023; Okumura and Zhao 2022; Lin et al. 2023). Since Coelotinae is species-rich in East Asia, our research has been continuously focused on the species diversity of Coelotinae from an integrative morphological and genetic perspective.

Upon examination of specimens collected from southern China and northern Vietnam, we suspected that they should belong to a new genus and five undescribed putative species. Therefore, morphological and phylogenetic analyses and comparisons with closely related species were carried out to confirm this. Here we report the results of these analyses, and describe in detail the new species and the genus erected to accommodate them.

## ﻿Material and methods

### ﻿Sampling and morphological examination

All specimens studied in this paper were collected from southern China and northern Vietnam and are deposited in the Institute of Zoology, Chinese Academy of Sciences (IZCAS). Specimens were examined with a LEICA M205 C stereomicroscope at IZCAS. Photos were taken with an Olympus C7070 wide zoom digital camera (7.1 megapixels) mounted either on an Olympus SZX12 dissecting microscope or on an Olympus BX51 compound microscope. Images from multiple focal ranges were combined using Helicon Focus v.6.80 photo stacking software. The epigyne and male palp were dissected for examination. The epigyne was treated in a warm 10% potassium hydroxide (KOH) solution. Images of the left male palp are illustrated. Measurements were obtained with a LEICA M205 C stereomicroscope and are given in mm. Eye diameters were measured as the maximum distance in either dorsal or frontal views. Leg measurements are given as follows: total length (coxa, trochanter, femur, patella, tibia, metatarsus, and tarsus). Terminology follows Wang (2003) and Li et al. (2018b, c). Abbreviations of eyes used in the text are as follows:

**ALE** anterior lateral eye;

**ALE–PLE** distance between ALE and PLE;

**AME** anterior median eye;

**AME–ALE** distance between AME and ALE;

**AME–AME** distance between AME and AME;

**AME–PME** distance between AME and PME;

**PLE** posterior lateral eye;

**PME** posterior median eye;

**PME–PLE** distance between PME and PLE;

**PME–PME** distance between PME and PME.

### ﻿Laboratory protocols and phylogenetic analyses

The DNA barcodes of the putative new species were obtained to test the species boundaries. A partial fragment of the mitochondrial cytochrome oxidase subunit I (*CO1*) gene was amplified and sequenced using the primers LCO1490-oono (5’-CWACAAAYCATARRGATATTGG-3’) and HCO2198-zz (5’-TAAACTTCCAGGTGACCAAAAAATCA-3’), following Zhao and Li (2017). GenBank accession numbers of *CO1* are listed separately in Table 1. The molecular dataset consisted of: *CO1* gene, histone 3 (*H3*) gene, NADH dehydrogenase subunit I (*ND1*) gene, *wingless* gene and the ribosomal RNA genes *12S*, *16S*, *18S*, and *28S*; in total eight genes of 77 species that were recently published, including 68 species in 33 known genera of Coelotinae (with 27 type species from different genera) as the ingroup, and four species of Ageleninae and Amaurobiidae as the outgroup (Zhao and Li 2017; Okumura and Zhao 2022; Li et al. 2023), alongside three novel sequences. GenBank accession numbers for all the above genes are shown in Suppl. material 1.

**Table 1.** Voucher specimen information.

Species	Voucher code	GenBank accession number	Sequence length	Collection localities
*B.daxi* sp. nov.	IZCAS-Ar44390 (LB084)	OQ721077	621 bp	Guilin, Guangxi, China
*B.pindong* sp. nov.	IZCAS-Ar44392 (LB172)	OQ721076	627 bp	Qingyuan, Guangdong, China
*B.tamdao* sp. nov.	IZCAS-Ar44393 (ZZ495)	KY778894	1194 bp	Vinh Phuc, Vietnam
*B.zhuping* sp. nov.	IZCAS-Ar44394 (ZZ124)	KY778886	613 bp	Kaili, Guizhou, China
*B.zuojiang* sp. nov.	IZCAS-Ar44396 (LB070)	OQ721078	906 bp	Chongzuo, Guangxi, China

Phylogenetic relationships were inferred using both maximum likelihood (ML) and Bayesian inference (BI). First, the best-fit partitioning schemes and models were selected for the RAxML and MrBayes analyses by using PartitionFinder v.2.1.1 (Lanfear et al. 2012). ML analysis was conducted in RAxML v.8.0.0 (Stamatakis 2006) using the substitution model GTRCAT for all partitions (partitioned by genes). A rapid bootstrap (‘-f a’) of 1000 replicate ML inferences was performed to search for the best-scoring ML tree and compute nodal support. BI analysis were performed in MrBayes v.3.2.2 (Ronquist and Huelsenbeck 2003) with posterior distributions estimated by Markov chain Monte Carlo (MCMC) sampling. The appropriate model was selected for each partition gene: the GTR+I+G model was favored for each partition, except that different models were selected for *H3* (HKY+I+G), *wingless* (SYM+I+G) and *18S* (K80+I+G). Two simultaneous runs with four MCMC chains were performed for 10 million generations to ensure that the average standard deviation of the split frequency was below 0.01 and to obtain a well-supported consensus tree. Then, ML analysis was also performed in IQ-TREE v.1.6.12 (Nguyen et al. 2015) by using ModelFinder function (-m MFP+MERGE) to select the best-fit model for each partition, and the option ‘-bb 1000’ to estimate the nodal support values.

## ﻿Results and discussion

The five species of the new genus share similar external genital morphology such as a long femur (more than three times longer than patella), a short patella (c. half the length of tibia), a bent tibia, the base of cymbium with one or two hypophyses, a wide embolus having a widest anterior, a large dorsal apophysis of conductor with a jagged margin; an epigyne lacking epigynal teeth, an anterior atrium located over the swell of epigyne and posterior epigynal sclerite between two swells of the epigyne, spermathecae small (shorter than 1/4 the length of copulatory ducts) and located posteriorly, close to each other, which anterior part fist-like.

**Figure 1.**
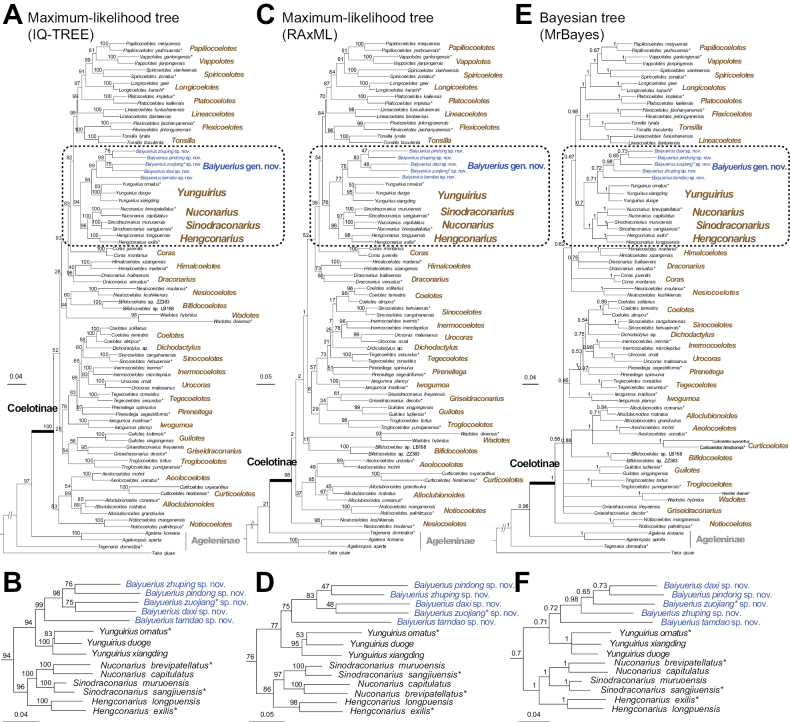
Phylogenetic trees **A, B** maximum likelihood (ML) trees obtained by using IQ-TREE **C, D**ML trees obtained by using RAxML **E, F** Bayesian trees obtained by using MrBayes. Support values for major nodes are shown. The scale bar corresponds to the expected number of substitutions per site. Asterisks express the type species of each genus.

Our phylogenetic analyses all infer similar tree topologies (Fig. 1) and strongly support *Baiyuerius* gen. nov. as a monophyletic clade (ML bootstrap = 100 and 95; BI posterior probability = 1.00). Geographically, species belonging to *Baiyuerius* gen. nov. are restricted to southern China and northern Vietnam (Fig. 8). Zoogeographic studies suggest that the genus-level distribution of coelotine spiders is regional, and the divergence and formation of these monophyletic genera are closely linked to geological and climatic events that occurred during the Neogene in Eurasia (Zhao and Li 2017; Zhao et al. 2020, 2022).

Based on these results, taking into account morphological comparisons, phylogenetic analyses, and zoogeographic considerations, *Baiyuerius* gen. nov. is established herein.

## ﻿Taxonomy

### ﻿Family Agelenidae C.L. Koch, 1837

**Subfamily Coelotinae F.O. Pickard-Cambridge, 1893**

#### ﻿Genus Baiyuerius

Taxon classificationAnimaliaAraneaeAgelenidae

Zhao, B. Li & S. Li gen. nov.

1D4D3AE7-FC64-5BF4-AF3B-D2DB6409C94F

https://zoobank.org/6E4A25C4-6D05-42E0-AEE2-D0106A932C23

**Figure 2.**
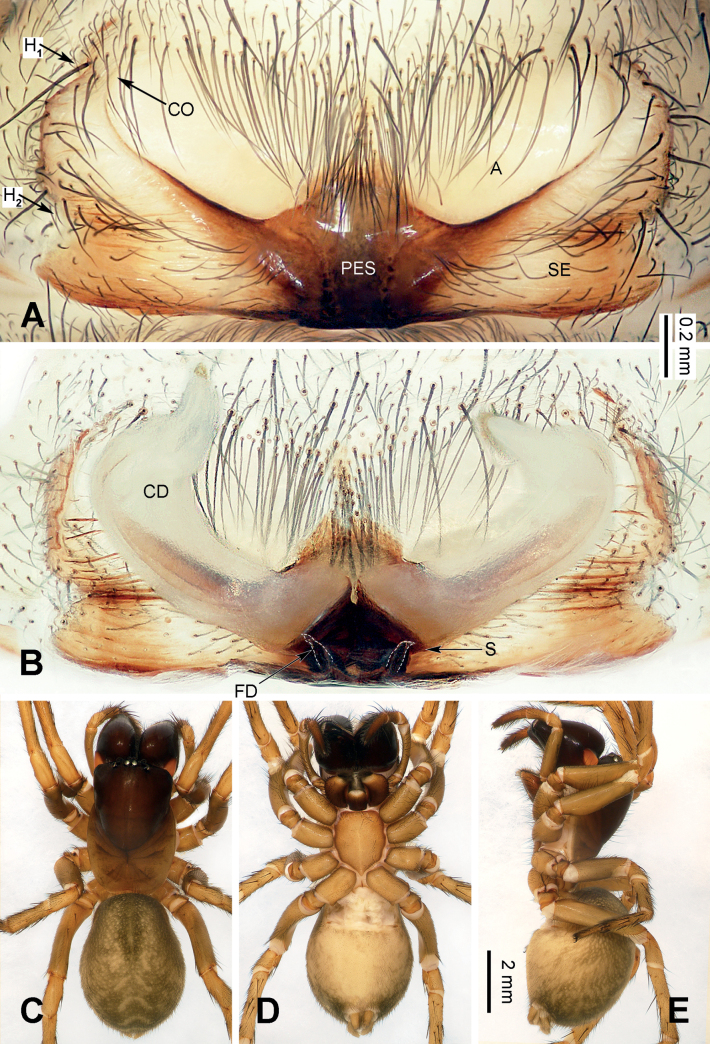
Epigyne and habitus of *Baiyueriusdaxi* sp. nov. **A** epigyne, ventral view **B** vulva, dorsal view **C** female habitus, dorsal view **D** female habitus, ventral view **E** female habitus, lateral view. Scale bar equal for **C–E**. Abbreviations: A = atrium; CD = copulatory duct; CO = copulatory opening; FD = fertilization duct (white dotted lines); H = hood; PES = posterior epigynal sclerite; S = spermatheca; SE = swell of epigyne.

**Figure 3.**
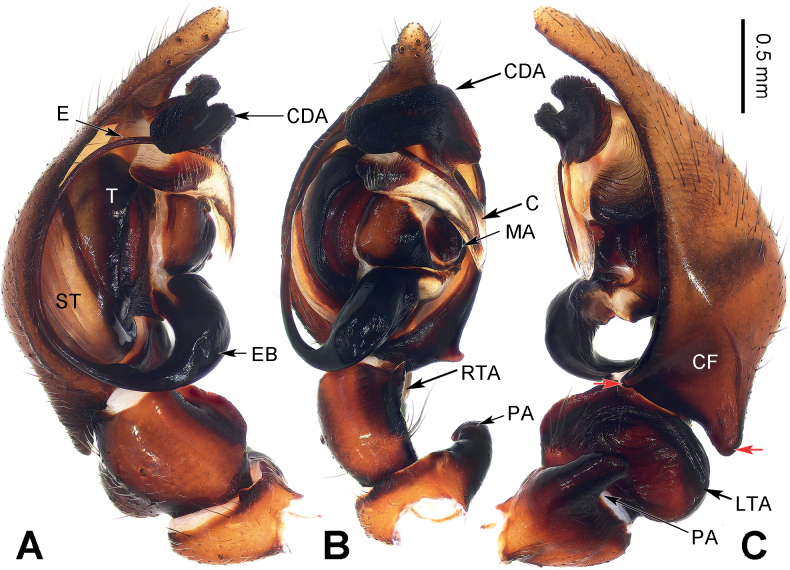
Left male palp of *Baiyueriuspindong* sp. nov. **A** prolateral view **B** ventral view **C** retrolateral view (red arrows = hypophyses of cymbium). Scale bar equal for **A–C**. Abbreviations: C = conductor; CDA = dorsal apophysis of conductor; CF = cymbial furrow; E = embolus; EB = embolic base; LTA = lateral tibial apophysis; MA = median apophysis; PA = patellar apophysis; RTA = retrolateral tibial apophysis; ST = subtegulum; T = tegulum.

**Figure 4.**
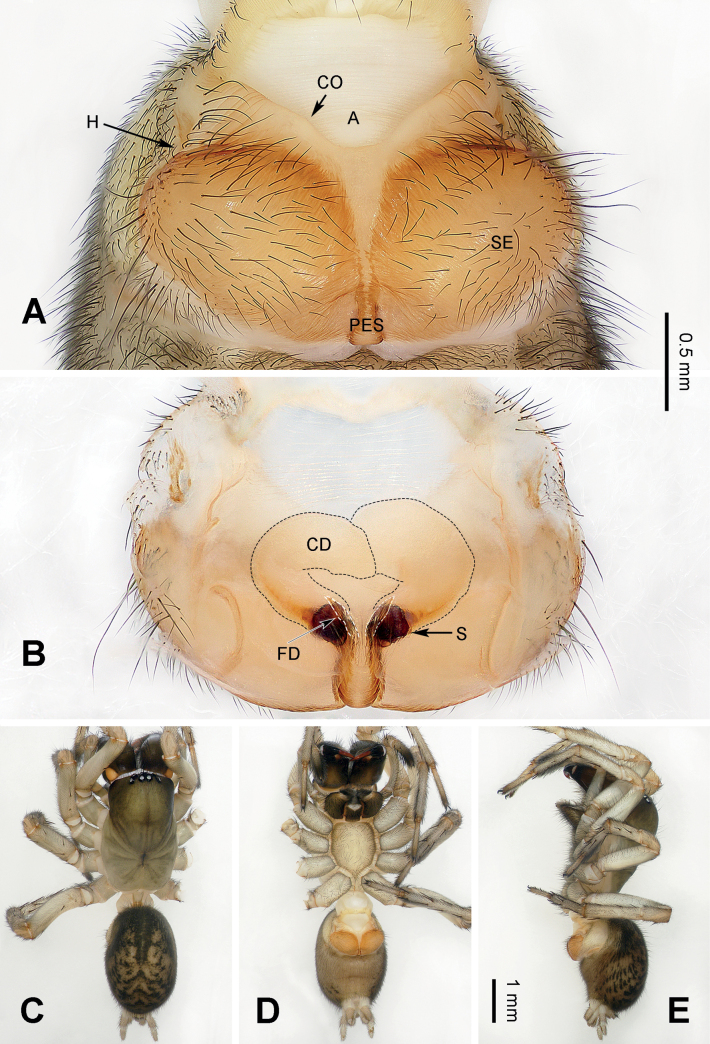
Epigyne and habitus of *Baiyueriustamdao* sp. nov. **A** epigyne, ventral view **B** vulva, dorsal view **C** female habitus, dorsal view **D** female habitus, ventral view **E** female habitus, lateral view. Scale bar equal for **C–E**. Abbreviations: A = atrium; CD = copulatory duct (black dotted lines); CO = copulatory opening; FD = fertilization duct (white dotted lines); H = hood; PES = posterior epigynal sclerite; S = spermatheca; SE = swell of epigyne.

**Figure 5.**
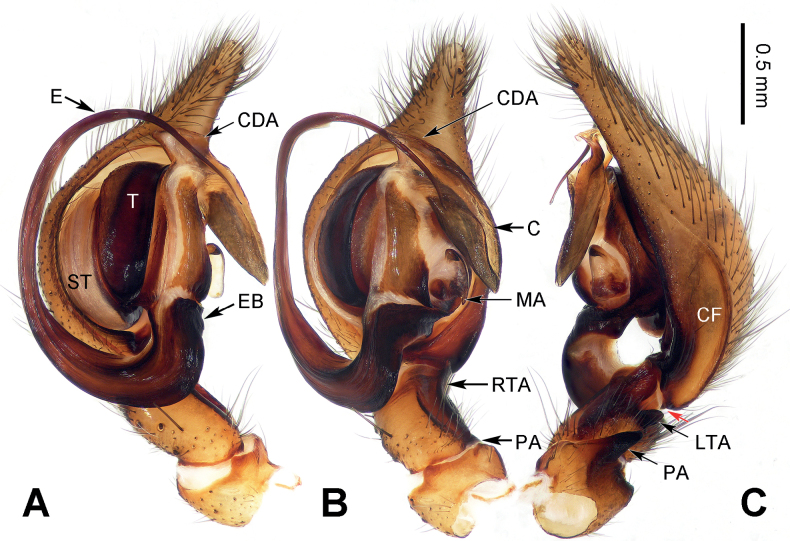
Left male palp of *Baiyueriuszhuping* sp. nov. **A** prolateral view **B** ventral view **C** retrolateral view (red arrow = hypophysis of cymbium). Scale bar equal for **A–C**. Abbreviations: C = conductor; CDA = dorsal apophysis of conductor; CF = cymbial furrow; E = embolus; EB = embolic base; LTA = lateral tibial apophysis; MA = median apophysis; PA = patellar apophysis; RTA = retrolateral tibial apophysis; ST = subtegulum; T = tegulum.

**Figure 6.**
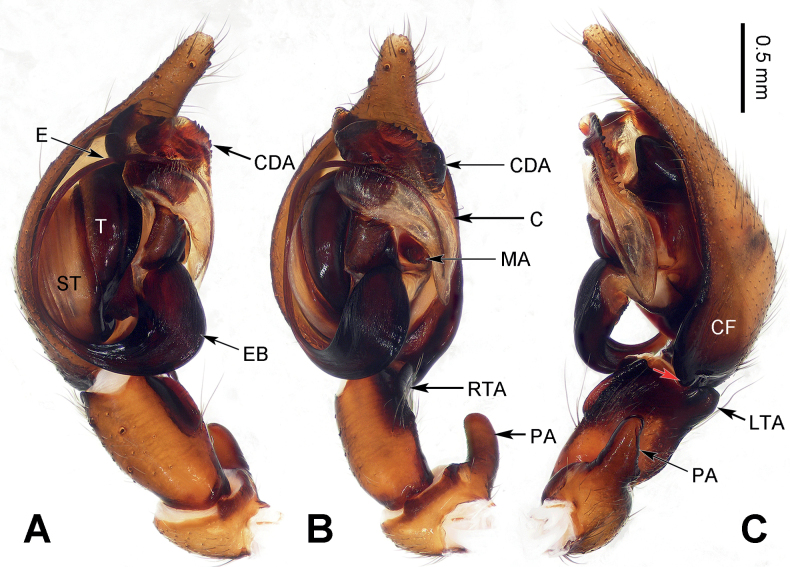
Left male palp of *Baiyueriuszuojiang* sp. nov. **A** prolateral view **B** ventral view **C** retrolateral view (red arrow: hypophysis of cymbium). Scale bar equal for **A–C**. Abbreviations: C = conductor; CDA = dorsal apophysis of conductor; CF = cymbial furrow; E = embolus; EB = embolic base; LTA = lateral tibial apophysis; MA = median apophysis; PA = patellar apophysis; RTA = retrolateral tibial apophysis; ST = subtegulum; T = tegulum.

**Figure 7.**
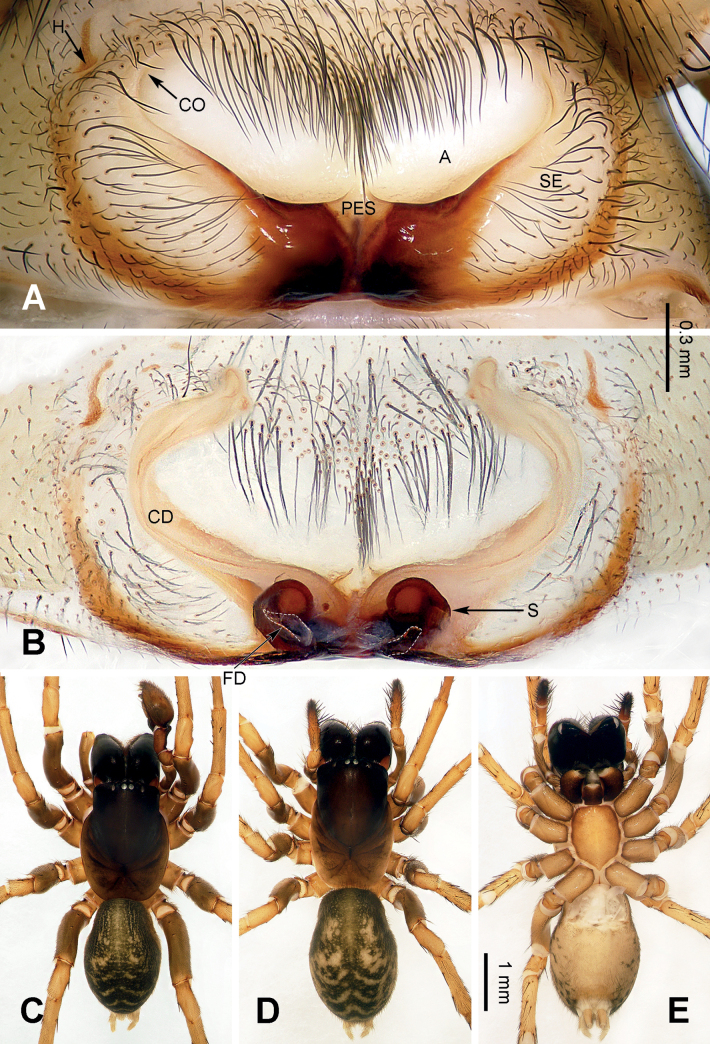
Epigyne and habitus of *Baiyueriuszuojiang* sp. nov. **A** epigyne, ventral view **B** vulva, dorsal view **C** male habitus, dorsal view **D** female habitus, dorsal view **E** female habitus, ventral view; Scale bar equal for **C–E**. Abbreviations: A = atrium; CD = copulatory duct; CO = copulatory opening; FD = fertilization duct (white dotted lines); H = hood; PES = posterior epigynal sclerite; S = spermatheca; SE = swell of epigyne.

**Figure 8.**
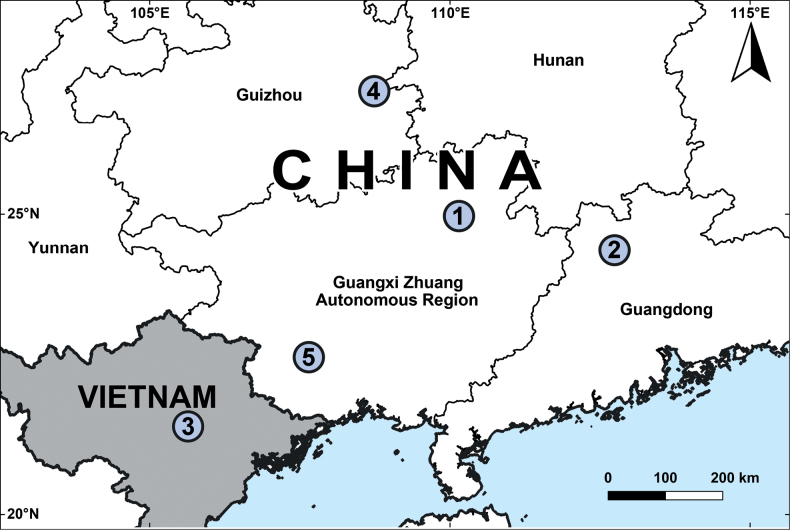
Localities of *Baiyuerius* species in China and Vietnam **1***B.daxi* sp. nov. **2***B.pindong* sp. nov. **3***B.tamdao* sp. nov. **4***B.zhuping* sp. nov. **5***B.zuojiang* sp. nov.

Figs 2 , 3 , 4 , 5 , 6 , 7 , 8

##### Type species.

*Baiyueriuszuojiang* Zhao, B. Li & S. Li, sp. nov., from Chongzuo, Guangxi Zhuang Autonomous Region, China.

##### Etymology.

The generic name is derived from the pinyin word “Baiyue”, referring to the Baiyue region where the new genus is distributed. Baiyue, a loose term dating back to the first millennium BC, was used to denote various populations who inhabited southern China and northern Vietnam. The postfix “-rius” refers to the postfix commonly used in the genera of the *Sinodraconarius* clade. The gender is masculine.

##### Diagnosis.

The morphological characteristics of *Baiyuerius* gen. nov. resemble those of *Yunguirius*, which is the closest genus to *Baiyuerius* gen. nov., by the dark color of the carapace, endites and labium; tibia longer than patella of male palp; dorsal apophysis of conductor large; embolus thick with swollen base; copulatory ducts membranous, arising posteriorly, along the contour of epigynal atrium. However, it can be distinguished from *Yunguirius* as follows: 1) the base of cymbium enlarged, with 1 or 2 hypophyses (Figs 3C, 5C, 6C) vs. without any hypophysis in *Yunguirius*; 2) an atrium located anteriorly and occupying less than or equal to 1/2 of the epigyne (Figs 2A, 4A, 7A) vs. located centrally and occupying more than 1/2 of the epigyne in *Yunguirius* (figs 2A, 3A, 4A in Li et al. 2023); and 3) the simple spermathecae (Figs 2B, 4B, 7B) vs. spermathecal heads long and continuous with its copulatory ducts in *Yunguirius* (figs 2B, 3B, 4B in Li et al. 2023).

##### Description.

Medium-sized, total lengths from 8.60 to 11.98. Carapace black turning brown or brown turning yellow-brown, pear-shaped, with longitudinal fovea and darker radial grooves; chelicerae as the same color as the anterior carapace, with three promarginal and two retromarginal teeth; endites and labium dark brown or grey, anteriorly white with black hairs; sternum brown or milk-white, longer than wide. Abdomen yellow-brown, covered with grey hairs, with two pairs of apodemes and four darker chevron-like markings. Spinnerets lighter than sternum in color. Leg formula 4 > 1> 2 > 3. Male palp: femur more than 3 times than patella, patella approx. half of tibia, patellar apophysis thick and enlarged, finger-like, longer than half of tibia and extending over patella, retrolateral tibial apophysis rectangular and lamellar, extending beyond tibia, lateral tibial apophysis of the same shape as patellar apophysis but thinner and shorter, median apophysis spoon-shaped, length of cymbial furrow c. 1/2 length of cymbium, with an enlarged base, the base of cymbium with 1 or 2 hypophyses, embolus widen and slightly elongated, anterior widest, dorsal apophysis of conductor large and in different shapes, in some cases bearing a jagged margin; Female epigyne: epigynal teeth absent, atrium located anteriorly, over the swell of epigyne, internally milk-white, occupying more than or equal to 1/4 of the female epigyne, posterior epigynal sclerite varying in shape and between two swollen parts of epigyne, copulatory ducts beginning at the posterior margin of epigyne, extended anteriorly, copulatory opening located anterolaterally, spermathecae small and located posteriorly, shorter than 1/4 the length of its copulatory ducts, anterior part fist-like, while its base close to each other, fertilization ducts originating from inside of spermathecae.

##### Distribution.

Guizhou and Guangdong Province, Guangxi Zhuang Autonomous Region, China and Vinh Phuc Province, Vietnam (Fig. 8).

#### ﻿ Baiyuerius daxi

Taxon classificationAnimaliaAraneaeAgelenidae

Zhao, B. Li & S. Li sp. nov.

899CC7F6-5A56-5304-8D59-FA741F3F3201

https://zoobank.org/CFB7093C-F38D-46A2-B5DF-1E109A8CE591

Figs 2 , 8

##### Type material.

***Holotype*** ♀ (IZCAS-Ar44390) (LB084): China: Guangxi Zhuang Autonomous Region: Guilin City: Yongfu County, Luojin Town, Daxi Village, Fushouyan Cave, 24.9704°N, 110.1463°E, elevation: 308 m, 4.I.2018, Z. Chen leg. ***Paratype***: 1♀ (IZCAS-Ar44391) (YX562): same town as holotype, Jinzhongshan Scenic Area, Yongfuyan Cave (a cave near Fushouyan cave), 24.9731°N, 110.1417°E, elevation: 236 m, 24.X.2019, Z. Chen leg.

##### Etymology.

The new species is named after the type locality, the Daxi Village; noun in apposition.

##### Diagnosis.

*Baiyueriusdaxi* sp. nov. resembles *B.zuojiang* sp. nov. by atrium glasses-shaped, outsides of posterior epigynal sclerite dark brown and copulatory ducts extending along the sclerotic margins of the atrium to its anterolateral margin. However, it can be distinguished from *B.zuojiang* as follows: 1) epigyne with two hoods (located anterolaterally and central laterally) (Fig. 2A) vs. only one hood (located anterolaterally) in *B.zuojiang* (Fig. 7A); 2) posterior epigynal sclerite pentagonal (Fig. 2A) vs. rhomboid in *B.zuojiang* (Fig. 7A); and 3) spermathecae extending anteriorly (Fig. 2B) vs. extending laterally in *B.zuojiang* (Fig. 7B).

##### Description.

**Female** (holotype) (Fig. 2C–E). Total length 11.98. Carapace 6.02 long, 3.51 wide. Abdomen 5.96 long, 4.29 wide. Eye sizes and interdistances: AME: 0.11, ALE: 0.16, PME: 0.14, PLE: 0.15; AME–AME: 0.06; AME–ALE: 0.09; AME–PME: 0.04; ALE–PLE: 0.04; PME–PME: 0.05; PME–PLE: 0.16. Leg measurements: I: 14.81 (1.38, 0.56, 4.02, 1.29, 3.03, 2.61, 1.92); II: 13.26 (1.28, 0.57, 3.68, 1.27, 2.26, 2.35, 1.85); III: 11.85 (1.09, 0.47, 3.04, 1.22, 2.03, 2.42, 1.58); IV: 15.58 (1.35, 0.59, 4.29, 1.36, 2.85, 3.15, 1.99). Leg formula 4 > 1> 2 > 3. Carapace black turning brown, chelicerae black, endites and labium brown, sternum, spinnerets, and legs yellow-brown. Female epigyne (Fig. 2A, B): atrium glasses-shaped, occupying half of epigyne, hoods located anterolaterally and central laterally, posterior epigynal sclerite near pentagonal, externally wrinkled and enlarged, copulatory ducts originating centrally and near posteriorly, extending first posteriorly then anteriorly, 4 times longer than wide, copulatory opening located anterolaterally, spermathecae coiled, touching each other, anterior 1/2 covered by its copulatory ducts, fertilization ducts separate from each other, c. 2.5 times longer than wide.

**Male.** Unknown.

##### Distribution.

Guangxi Zhuang Autonomous Region, China (Fig. 8).

#### ﻿ Baiyuerius pindong

Taxon classificationAnimaliaAraneaeAgelenidae

Zhao, B. Li & S. Li sp. nov.

E1E650EB-88C3-5415-A694-B9E6D280D05D

https://zoobank.org/8F9E7DC6-91B2-49B6-A0B2-DE7657E52179

Figs 3 , 8

##### Type material.

***Holotype*** ♂ (IZCAS-Ar44392) (LB172): China: Guangdong Province: Qingyuan City: Yangshan County, Shuikou Ancient Town, Pindong Village, 24.3987°N, 112.7433°E, elevation: 553 m, 8.I.2019, Z. Zhao and Z. Chen leg.

##### Etymology.

The new species is named after the type locality, the Pindong Village; noun in apposition.

##### Diagnosis.

*Baiyueriuspindong* sp. nov. can be distinguished from the other congeners of this new genus as follows: 1) cymbial furrow less than 1/2 the length of cymbium (Fig. 3C) vs. more than 1/2 in others (Figs 5C, 6C); 2) cymbial base with two hypophyses (Fig. 3C) vs. only one in others (Figs 5C, 6C); and 3) lateral tibial apophysis concave and large, wider than long (Fig. 3C) vs. finger-like, longer than wide in others (Figs 5C, 6C).

##### Description.

**Male** (holotype). Total length 10.06. Carapace 5.42 long, 3.53 wide. Abdomen 4.64 long, 2.96 wide. Eye sizes and interdistances: AME: 0.12, ALE: 0.15, PME: 0.15, PLE: 0.15; AME–AME: 0.06; AME–ALE: 0.09; AME–PME: 0.07; ALE–PLE: 0.04; PME–PME: 0.04; PME–PLE: 0.15. Leg measurements: I: 17.13 (2.01, 0.76, 4.13, 1.74, 3.06, 3.17, 2.26); II: 15.78 (1.89, 0.75, 3.59, 1.73, 2.76, 3.14, 1.92); III: 13.52 (1.57, 0.74, 3.14, 1.34, 1.92, 3.12, 1.69); IV: 17.96 (1.78, 0.81, 4.43, 1.76, 3.14, 4.12, 1.92). Leg formula 4 > 1> 2 > 3. Carapace black turning dark brown, chelicerae, endites, and labium dark brown, sternum brown, longer than wide, spinnerets yellow-brown, legs dark brown turning yellow-brown. Male palp (Fig. 3): femur long, c. 4 times longer than wide, tibia long, c. 2 times longer than wide, patella short, length only 1/3 of width, patellar apophysis dark brown, c. 3 times longer than wide, extending over half of tibia, with a blunt and bent distal end, retrolateral tibial apophysis originating from 1/3 of tibia, lateral tibial apophysis enlarged, human-ear-like, median apophysis spoon-shaped, cymbial furrow subequal to 1/2 the length of cymbium, cymbial base with two hypophyses, embolus originating at a 7 o’clock, widen, narrowing in the second half, then wrapped by conductor, embolic base black, 2 times longer than wide, conductor translucent, with wrinkles, while its margin jagged and transparent, dorsal apophysis of conductor black and strongly expanded, leaf-like, with a jagged margin.

**Female.** Unknown.

##### Distribution.

Guangdong Province, China (Fig. 8).

#### ﻿ Baiyuerius tamdao

Taxon classificationAnimaliaAraneaeAgelenidae

Zhao, B. Li & S. Li sp. nov.

CD538138-03B2-52B5-9075-01CA4D608761

https://zoobank.org/895A75E8-EDCE-41F0-A8B0-66E12C2112A5

Figs 4 , 8

##### Type material.

***Holotype*** ♀ (IZCAS-Ar44393) (ZZ495): Vietnam: Vinh Phuc Province: Tam Dao National Park (field), 21.4720°N, 105.6364°E, elevation: 1023 m, 31.X.2012, H. Zhao and Z. Chen leg.

##### Etymology.

The new species is named after the type locality, the Tam Dao National Park; noun in apposition.

##### Diagnosis.

*Baiyueriustamdao* sp. nov. can be distinguished from the other congeners of this genus as follows: 1) swell of epigyne as twice as atrium, spherical and uniformly yellow (Fig. 4A) vs. as the same size as atrium in others, long-eggplant-shaped, with brown margin in others (Figs 2A, 7A); 2) posterior epigynal sclerite near trapezoidal (Fig. 4A) vs. pentagonal (in *B.daxi*) or near rhomboid (in *B.zuojiang*) (Figs 2A, 7A); and 3) copulatory ducts extending anteriorly, anterior overlapping to each other (Fig. 4B) vs. extending along the sclerotic margins of the atrium and separated anteriorly in others (Figs 2B, 7B).

##### Description.

**Female** (holotype) (Fig. 4C–E). Total length 10.94. Carapace 5.86 long, 3.61 wide. Abdomen 5.08 long, 3.34 wide. Eye sizes and interdistances: AME: 0.11, ALE: 0.23, PME: 0.22, PLE: 0.26; AME–AME: 0.05; AME–ALE: 0.14; AME–PME: 0.09; ALE–PLE: 0.02; PME–PME: 0.06; PME–PLE: 0.23. Leg measurements: I: 15.25 (0.96, 0.42, 3.81, 1.69, 3.42, 2.80, 2.15); II: 13.63 (0.83, 0.36, 3.74, 1.60, 2.72, 2.41, 1.97); III: 11.18 (0.79, 0.32, 3.12, 1.53, 2.03, 1.96, 1.43); IV: 15.47 (0.98, 0.38, 4.31, 1.83, 3.19, 2.93, 1.85). Leg formula 4 > 1> 2 > 3. Carapace brown turning yellow-brown, chelicerae, endites, and labium grey, sternum, spinnerets, and legs milk-white, legs covered with grey hairs. Female epigyne (Fig. 4A, B): atrium inverted triangular, occupying 1/4 of epigyne, epigynal hood located located central laterally, posterior epigynal sclerite near trapezoidal, c. 3 times longer than wide, swell of epigyne near spherical and uniformly yellow, copulatory ducts originating centrally and near posteriorly, located centrally, extending anteriorly then curved inward, U-shaped, copulatory opening located centrally, on both sides of the midline, spermathecae coiled, fist-like, c. 1/6 the length of copulatory ducts, fertilization ducts slender and transparent, 5 times longer than wide.

**Male.** Unknown.

##### Distribution.

Vinh Phuc Province, Vietnam (Fig. 8).

#### ﻿ Baiyuerius zhuping

Taxon classificationAnimaliaAraneaeAgelenidae

Zhao, B. Li & S. Li sp. nov.

CA5FF662-BF30-51AE-9308-70F38FEFEE36

https://zoobank.org/E4FC3DC0-BEA4-4C11-BB6F-F701D6516D02

Figs 5 , 8

##### Type material.

***Holotype*** ♂ (IZCAS-Ar44394) (ZZ124): China: Guizhou Province: Kaili City: Zhenyuan County, Yangping Town, Zhuping Village, Zhangjiawan Cave, 27.0528°N, 108.7406°E, elevation: 578 m, 17.XII.2011, Z. Zha and Z. Chen leg.

##### Etymology.

The new species is named after the type locality, the Zhuping Village; noun in apposition.

##### Diagnosis.

*Baiyueriuszhuping* sp. nov. can be distinguished from all other congeners in this genus as follows: 1) dorsal apophysis of conductor small and light (Fig. 5A–C) vs. large and dark in others (Figs 3A–C, 6A–C); 2) the margin of conductor without any jags (Fig. 5A–C) vs. jagged in others (Figs 3A–C, 6A–C); and 3) distal end of patellar apophysis pointed (Fig. 5C) vs. blunt in others (Figs 3C, 6C).

##### Description.

**Male** (holotype). Total length 9.51. Carapace 4.93 long, 2.86 wide. Abdomen 4.58 long, 3.15 wide. Eye sizes and interdistances: AME: 0.11, ALE: 0.14, PME: 0.13, PLE: 0.13; AME–AME: 0.03; AME–ALE: 0.06; AME–PME: 0.05; ALE–PLE: 0.04; PME–PME: 0.04; PME–PLE: 0.09. Leg measurements: I: 12.40 (1.23, 0.41, 2.91, 0.92, 2.74, 2.54, 1.65); II: 11.48 (1.21, 0.38, 2.98, 0.92, 2.14, 2.21, 1.64); III: 10.17 (1.19, 0.34, 2.61, 0.83, 1.81, 2.03, 1.36); IV: 13.06 (1.32, 0.51, 3.18, 0.95, 2.81, 2.68, 1.61). Leg formula 4 > 1> 2 > 3. Carapace black turning dark brown, chelicerae, endites, and labium dark brown, sternum brown, longer than wide, spinnerets yellow-brown, legs dark brown turning yellow-brown. Male palp (Fig. 5): femur long, less than 5 times longer than wide, patella c. 1/2 the length of tibia, patellar apophysis brown turning dark brown and with a pointed distal end, more than 2 times longer than wide, extending over 2/3 of tibia, retrolateral tibial apophysis originating from the base of tibia, lateral tibial apophysis finger-like, pointed to posterolaterally, median apophysis with three black lobes, cymbial furrow c. 1/2 the length of cymbium, cymbial base with a hypophysis, embolus originating at a 7 o’clock, first 2/5 widen, and then narrowing and wrapped by conductor, embolic base concave and dark brown, 2 times wider than long, conductor membranous and yellow-brown, with a smooth and wrinkled surface and flat margin, dorsal apophysis of conductor translucent and square.

**Female.** Unknown.

##### Distribution.

Guizhou Province, China (Fig. 8).

#### ﻿ Baiyuerius zuojiang

Taxon classificationAnimaliaAraneaeAgelenidae

Zhao, B. Li & S. Li sp. nov.

250AA5BA-89CC-55F2-ADB4-F9F68495E688

https://zoobank.org/BEF10254-61A8-4218-85F0-46E5572D5711

Figs 6 , 7 , 8

##### Type material.

***Holotype*** ♂ (IZCAS-Ar44395) (LB070): China: Guangxi Zhuang Autonomous Region: Chongzuo City: Jiangzhou District, Tuolu Town, Zuojiang Overseas Chinese Farm, an unnamed cave, 22.6155°N, 107.6494°E, elevation: 107 m, 12.XII.2017, Z. Chen leg. ***Paratypes***: 1♂4♀♀ (IZCAS-Ar44396–Ar44400) (LB070): same data as holotype.

##### Etymology.

The new species is named after the type locality, the Zuojiang Overseas Chinese Farm; noun in apposition.

##### Diagnosis.

The males of *Baiyueriuszuojiang* sp. nov. resemble those of *B.pindong* sp. nov. by the margin of conductor jagged and dorsal apophysis large, patellar apophysis with a blunt and bent distal end; resemble those of *B.zhuping* sp. nov. by cymbial base with one hypophysis, lateral tibial apophysis finger-like. However, it can be distinguished from them by 1) median apophysis coiled and jagged (Fig. 6B) vs. without any jags and flat (in *B.pindong*) or with three lobes (in *B.zhuping*) (Figs 3B, C, 5B, C). The females of *B.zuojiang* sp. nov. resemble those of *B.daxi* sp. nov. by glasses-shaped atrium, and copulatory ducts extending first posteriorly then anteriorly, along the sclerotic margins of the atrium, anterior separated from each other. However, it can be distinguished from *B.daxi* as follows: 1) atrium occupying 1/3 of the epigyne (Fig. 7A) vs. half of epigyne in *B.daxi* (Fig. 2A); 2) posterior epigynal sclerite rhomboid, longer than wide (Fig. 7A) vs. pentagonal, as the same length as its width in *B.daxi* (Fig. 2A); and 3) copulatory ducts originating posteriorly, from the dorsal spermathecae then extending anteriorly (Fig. 7B) vs. originating centrally and near posteriorly, from the ventral spermathecae then extending anteriorly (Fig. 2B).

##### Description.

**Male** (holotype) (Fig. 7C). Total length 8.62. Carapace 4.59 long, 2.93 wide. Abdomen 4.03 long, 4.58 wide. Eye sizes and interdistances: AME: 0.11, ALE: 0.14, PME: 0.12, PLE: 0.15; AME–AME: 0.04; AME–ALE: 0.07; AME–PME: 0.04; ALE–PLE: 0.02; PME–PME: 0.02; PME–PLE: 0.11. Leg measurements: I: 12.76 (1.36, 0.46, 3.34, 1.07, 2.52, 2.42, 1.59); II: 12.29 (1.19, 0.44, 3.32, 1.03, 2.06, 2.47, 1.78); III: 10.26 (1.12, 0.41, 2.62, 1.04, 1.57, 2.02, 1.48); IV: 13.64 (1.12, 0.46, 3.44, 1.16, 2.48, 3.26, 1.72). Leg formula 4 > 1> 2 > 3. Carapace black turning dark brown, chelicerae, endites, and labium dark brown, sternum brown, longer than wide, spinnerets yellow-brown, legs dark brown turning yellow-brown. Male palp (Fig. 6): femur long, more than 4 times longer than wide, patella c. 1/4 of its bent tibia, patellar apophysis brown and with a blunt and bent distal end, 2.5 times longer than wide, extending over half of tibia, pointed to posterolaterally, retrolateral tibial apophysis originating from half of tibia, lateral tibial apophysis finger-like, distal end pointed to ventrally, median apophysis coiled to spoon-shaped, its anterior margin jagged, cymbial furrow c. 3/5 length of cymbium, cymbial base with a hypophysis, embolus dark brown turning black, originating at a 6 o’clock, first 1/4 widen, then narrowing and wrapped by conductor, embolic base 2 times longer than wide, conductor translucent, with a jagged margin, wrinkles and a membrane, dorsal apophysis translucent and brown, covered by the jagged membrane.

**Female.** (IZCAS-Ar44398) (Fig. 7D, E). Total length 9.58. Carapace 4.89 long, 2.77 wide. Abdomen 4.69 long, 3.01 wide. Eye sizes and interdistances: AME: 0.10, ALE: 0.15, PME: 0.14, PLE: 0.13; AME–AME: 0.06; AME–ALE: 0.08; AME–PME: 0.04; ALE–PLE: 0.04; PME–PME: 0.04; PME–PLE: 0.17. Leg measurements: I: 11.47 (0.77, 0.39, 3.38, 1.09, 2.22, 2.13, 1.49); II: 10.51 (0.63, 0.39, 2.74, 1.03, 2.10, 1.97, 1.65); III: 8.41 (0.58, 0.35, 2.24, 0.82, 1.21, 1.84, 1.37); IV: 11.78 (0.68, 0.38, 3.13, 1.13, 2.43, 2.69, 1.34). Leg formula 4 > 1> 2 > 3. Carapace black turning dark brown, chelicerae black, endites and labium dark brown, sternum brown, spinnerets and legs yellow-brown. Female epigyne (Fig. 7A, B): atrium glasses-shaped, occupying 1/3 of epigyne, with sclerotized lateral margins, epigynal hood located central laterally, posterior epigynal sclerite rhomboid, anterior of the lateral margin brown, while posterior of the lateral margin black, externally enlarged, milk-white, copulatory ducts originating posteriorly and extending anteriorly, c. 6.5 times longer than wide, spermathecae c. 1/4 of the length of copulatory ducts, spermathecal base close to each other, while anteriorly fist-like, fertilization ducts transparent, pointed to laterally, c. 3.5 times longer than wide.

##### Distribution.

Guangxi Zhuang Autonomous Region, China (Fig. 8).

## Supplementary Material

XML Treatment for Baiyuerius

XML Treatment for Baiyuerius daxi

XML Treatment for Baiyuerius pindong

XML Treatment for Baiyuerius tamdao

XML Treatment for Baiyuerius zhuping

XML Treatment for Baiyuerius zuojiang

## ﻿Additional information

### Author ORCIDs

Zhe Zhao https://orcid.org/0000-0002-0781-0204

Bing Li https://orcid.org/0000-0002-7106-4680

Xiaoqing Zhang https://orcid.org/0000-0003-0687-9266

Francesco Ballarin https://orcid.org/0000-0003-1417-2519

Dinh-Sac Pham https://orcid.org/0000-0001-8594-5270

### Data availability

All of the data that support the findings of this study are available in the main text or Supplementary Information.

## Supplementary materials

Supplementary material 1 The molecular dataset comprised eight genes of 77 species Data type GenBank accession numbers https://binary.pensoft.net/file/856274This dataset is made available under the Open Database License (http://opendatacommons.org/licenses/odbl/1.0/). The Open Database License (ODbL) is a license agreement intended to allow users to freely share, modify, and use this Dataset while maintaining this same freedom for others, provided that the original source and author(s) are credited.Zhe Zhao
